# Female With Shortness of Breath

**DOI:** 10.1016/j.acepjo.2025.100206

**Published:** 2025-06-17

**Authors:** Emma Alley, Garrett Stoltzfus, Eric Melnychuk

**Affiliations:** 1 Department of Emergency Medicine, Geisinger Medical Center, Danville, Pennsylvania, USA; 2 Department of Critical Care Medicine, Geisinger Medical Center, Danville, Pennsylvania, USA

**Keywords:** Bochdalek, hernia

## 1 Case

A 31-year-old female presented to the emergency department with shortness of breath. She noted that her shortness of breath was progressive over the last few months, and denied cough, congestion, and fever. Vital signs on arrival included pulse rate of 129 beats per minute, respiratory rate of 34 breaths per minute, and SpO_2_ of 50% on room air. Examination showed near complete absence of breath sounds on the left thorax. Computed tomography (CT) of her chest was obtained (Fig).

**Figure:**
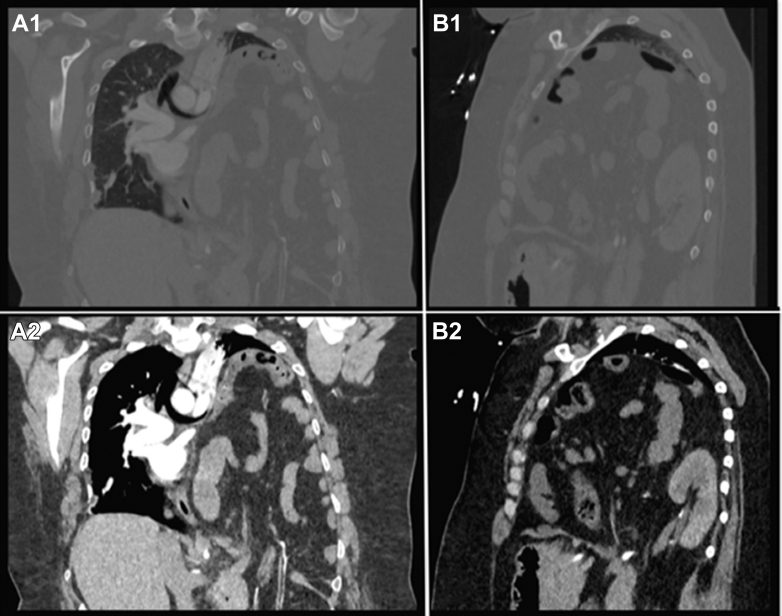
The images are obtained during a computed tomography pulmonary embolism study. Images marked with an “A” and “B” show the coronal and sagittal view of the patient’s chest, respectively. Images marked with a “1” and “2” are in the lung and abdominal view, respectively. The images show a chronic Bochdalek hernia. Near-complete atelectasis of the left lung is best seen in images A1 and B1. Image A2 demonstrates herniation of the visceral fat, bowels, and vascular structures across the diaphragm. Image B2 demonstrates the posterior diaphragmatic defect resulting in herniation of the bowel and the left kidney.

## 2 Diagnosis: Bochdalek Hernia

The patient was ultimately found to have severe atelectasis from compression of the lung by a left-sided congenital diaphragmatic hernia, also known as a Bochdalek hernia. Congenital diaphragmatic hernias are the leading cause of congenital hernias and result from a posterolateral diaphragmatic defect.^1^ Typically identified in the neonatal period, these hernias tend to be repaired early due to risk of pulmonary hypoplasia, pulmonary hypertension, or incarceration of abdominal contents. Congenital diaphragmatic hernias are rarely found in adults; however, when found show 69% experience pain, 39% obstruction, and 37% pulmonary symptoms.^2^ Bochdalek hernias occur 78% of the time on the left and 55% of occurrences are in males.^2^ CT is the most specific imaging modality for diagnosis, though Bochdalek hernias can be identified on x-ray, ultrasound, and magnetic resonance imaging.^2^ Treatment involves surgery in symptomatic adults, and the need for additional repair is common. Mortality is seen in 2.7% to 4.4% of cases.^1^ Ultimately, this patient underwent patch repair with improvement of her symptoms. As of writing this article, she has not had a recurrence of her hernia.

## Funding and Support

By *JACEP Open* policy, all authors are required to disclose any and all commercial, financial, and other relationships in any way related to the subject of this article as per ICMJE conflict of interest guidelines (see www.icmje.org). The authors have stated that no such relationships exist.

## Conflict of Interest

E. Melnychuk is an editor for the *Journal of the American College of Emergency Physicians Open*. The other authors have affirmed they have no conflicts of interest to declare.
